# Tuning the Pore Structures of Organosilica Membranes for Enhanced Desalination Performance via the Control of Calcination Temperatures

**DOI:** 10.3390/membranes10120392

**Published:** 2020-12-03

**Authors:** Rong Xu, Qian Liu, Xiuxiu Ren, Peng Lin, Jing Zhong

**Affiliations:** Jiangsu Key Laboratory of Advanced Catalytic Materials and Technology, School of Petrochemical Engineering, Changzhou University, Changzhou 213164, China

**Keywords:** membrane, organosilica, pore structure, desalination, calcination temperature

## Abstract

Microporous organosilica membranes based on 1,2-bis(triethoxylsilyl)ethane (BTESE) were fabricated via an acid-catalyzed sol-gel technique. In the preparation process, the calcination temperature plays a significant role in structural and surface properties of the organosilica networks. With an increase in calcination temperature, the surface hydrophilicity decreased due to the enhanced condensation of Si-OH groups in the networks. N_2_ adsorption results suggest that the pore structures of BTESE membranes was clearly dependent on the calcination temperature. The pore sizes of the membranes were quantitatively determined by using the Normalized Knudsen-based permeance (NKP) model. In pervaporation tests, the membranes with higher calcination temperatures showed higher salt rejections and lower water permeances, which was attributed to the changes in pore size and surface chemistry of pore walls. The BTESE membranes calcined at 200 °C exhibited superior hydrothermal stability in temperature cycles up to 70 °C and high reproducibility in concentration cycles with NaCl concentrations of 0.2–13 wt%, showing great promise for desalination applications of high-salinity water.

## 1. Introduction

The desalination of seawater or brackish water offers a steady supply of clean, fresh water for continuously growing populations. The major desalination technologies currently in use are thermal-driven distillation and membrane-based reverse osmosis (RO), with RO accounting for more than 50% of the installed capacity [1,2]. However, in order to push water to pass through a RO membrane, a high pressure of 5–8 MPa must be applied to overcome the osmotic pressure of seawater. Moreover, the commercial polyamide-based RO membranes are prone to biofouling, which severely diminishes their separation performance [3]. To address these challenges of RO, alternative desalination technologies such as membrane distillation [4], electrodialysis [5], and forward osmosis [6] have been proposed.

Pervaporation (PV), as an energy-efficient membrane separation technology, has been extensively used for the separation of organic/water mixtures and organic/organic mixtures [7]. Recently, many research efforts are focusing on PV in hopes of applying this technology to desalination [8,9]. Compared with the RO process, PV has the advantage of nearly 100% salt rejection and the energy consumption is essentially independent of the feed salinity. This feature makes PV particularly suited for desalination of high-salinity water, such as produced water from oil or gas production [10]. Additionally, PV is typically carried out at 30–90 °C, which is lower than conventional distillation. Thus, the low-grade heat energy (e.g., waste heat from power plants or geothermal energy) can be utilized, reducing the external energy use of the process [11].

Recently, various membranes have been explored for PV desalination by using different types of materials, such as poly(vinyl alcohol) (PVA) [12], graphene oxide (GO) [13], zeolitic imidazolate framework (ZIF) [14], and zeolites [15,16,17]. The salt rejection of these membranes is more than 98%, but they typically possess low water permeance and/or membrane stability in desalination of high-salinity water. Microporous amorphous silica with pore size of 2–5 Å is another promising candidate for the application in molecular separation. However, a low hydrothermal stability limits its application in a water-containing system. A significant breakthrough in hydrothermal stability came with the development of organically bridged silica, in which the less stable siloxane bonds (Si–O–Si) were partially replaced by hydrolytically stable organic bridges (Si–C_x_–Si) [18,19]. Castricum et al. [18] developed the organosilica membranes via co-condensation of 1,2-bis(triethoxysilyl)ethane (BTESE) and methyltriethoxysilane (MTES). The prepared membranes delivered a quite stable performance over 2 years for pervaporation dehydration of 95 wt% n-butanol. Afterwards, the applications of BTESE membranes have expanded to gas separation, RO and nanofiltration (NF) processes [20,21,22]. The only weakness of BTESE membranes was the relatively low water permeance, mainly due to the confined pore size and the hydrophobic nature of the networks, which would not allow water to rapidly transport through the membrane. Thus, pore structures of the organosilica networks must be altered in order to achieve a high water permeation rate.

The Sol-gel (SG) technique is widely used to prepare silica-based membranes [23]. In the SG process, the alkoxysilanes are hydrolyzed with water to form Si-OH and then condensed to form Si-O-Si networks, as follows:

≡Si–OEt + H_2_O ⇔ ≡Si–OH + EtOH (1)

≡Si–OH + ≡Si–OH ⇔ ≡Si–O–Si≡ + H_2_O (2)

≡Si–OEt + ≡Si–OH ⇔ ≡Si–O–Si≡ + EtOH(3)

The hydrolysis conditions such as water/alkoxysilane ratios in the first reaction have been investigated extensively [24,25], but the condensation conditions in the reactions of (2) and (3) are rarely studied. The calcination temperature is one of the key factors of the condensation reactions. Kanezashi et al. reported a decrease from 0.385 to 0.347 nm in pore size of silica membranes with increasing the calcination temperatures from 550 to 750 °C [26]. Qi et al. fabricated BTESE membranes with pore size of 0.362–0.454 nm by firing at 400–600 °C together with a predesigned heating rate and dwelling time [27]. However, these membranes calcined at high temperatures are not appropriate for the applications in water desalination, because the silica networks with small pore size and less silanol groups would hinder the rapid transport of water molecules.

In the present study, BTESE-derived organosilica membranes were fabricated at low calcination temperatures of 100–300 °C and applied to pervaporation of aqueous solutions with varying salinity levels. In particular, the effect of calcination temperature on structural and surface properties of the organosilica networks was extensively investigated. Various characterizations were performed to provide information about pore structure and surface chemistry of the BTESE membranes. In addition, the desalination performances and transport properties for these membranes with different calcination temperatures were discussed.

## 2. Experimental

### 2.1. Sol Synthesis

The precursors of BTESE was first dissolved into ethanol, then water and catalyst HCl were added under continuous stirring. The molar ratio was BTESE:H_2_O:HCl = 1:60:0.3, and the weight percent of BTESE was kept at 5 wt% by using ethanol addition. The solution was stirred for 2 h at 40 °C to develop sols by the hydrolysis and polymerization reaction. Finally, the sols were put in the refrigerator at 0 °C to keep them stable.

### 2.2. Membrane Preparation

The porous α-alumina plates (average pore size: 200 nm, porosity: 35%) were used as the supports. First, α-alumina nanoparticles (average particle size: 200 nm) were deposited on the substrate using the SiO_2_-ZrO_2_ sols (molar ratio of Si/Zr = 1/1, 2 wt%) as a binder, and the substrate was fired at 550 °C for 30 min in air. Then, SiO_2_-ZrO_2_ sols with concentration of 0.5 wt% were coated onto the substrate using hot-coating method to form intermediate layers. The substrate was preheated up to about 180 °C, followed by quickly contacting the substrate with a wet cloth with the SiO_2_-ZrO_2_ sols. Subsequently, the substrate was fired at 550 °C for 15 min under air atmosphere. This hot-coating and calcination procedures were repeated 6–10 times to remove large pores that might cause pinholes in the separation layer. Finally, the BTESE-derived separation layer was fabricated by wipe-coating of 0.5 wt% BTESE sols (diluted by ethanol) onto the intermediate layers, followed by calcination for 20 min at 100, 200, or 300 °C in air, and denoted as BTESE-100, BTESE-200, and BTESE-300 membranes, respectively.

### 2.3. Characterization

A Fourier transform infrared spectrometer (FT-IR, Bruker TENSOR-27, Thermal Energy Corporation, Houston, TX, USA) was applied to test the chemical structure of BTESE-derived films at different calcination temperatures. The morphology of the membrane was examined using field emission scanning electron microscopy (FE-SEM, Zeiss SUPRA-55, Zeiss AG, Oberkochen, Germany) with an acceleration voltage of 5 kV. The BTESE-derived gel powders were examined by using nitrogen adsorption at 77 K (Micromeritics ASAP 2020 volumetric gas adsorption analyzer, Micromeritics Corporation, Norcross, GA, USA). The specific surface area was calculated by using the Brunauer-Emmett-Teller (BET) method in a relative pressure range of P/P_0_ = 0.01–0.25. The micropore volume was estimated using the *t*-plot method. The pore size distribution was obtained through the analysis of the adsorption branch of nitrogen isotherms using the MP method (Mikhail’s micropore analytical method). The cross-sectional composition the membrane after a long-term desalination process was analyzed by using energy dispersive X-ray spectroscopy (EDX, Zeiss SUPRA-55, Zeiss AG, Oberkochen, Germany).

### 2.4. Membrane Performance

Single gas permeation experiment was carried out at 100 °C using He, H_2_, N_2_, C_3_H_8_, and SF_6_. Prior to measure, the membrane was first dried for 10 h in a He flow rate of 20 m∙min^−1^ at 100 °C to remove the adsorbed water from the membrane pores. The permeate steam was maintained at atmospheric pressure, and the pressure drop across the membrane was kept at 100 kPa.

PV desalination experiments were conducted using a NaCl aqueous solution with concentrations from 0.2 wt% to 13 wt% to simulate the typical salinity of brackish (0.2–1 wt%), sea (~3.5 wt%), and brine (~7–15 wt%) waters. The experiments were carried out using a typical PV testing apparatus, as previously described [28]. The feed solution was continuously circulated using a variable speed peristaltic pump. The permeate water vapor was collected in cold traps using liquid nitrogen as cooling agents, and the permeate pressure was kept below 300 Pa by a vacuum pump.

The solution-diffusion (SD) is the most widely accepted model to describe the mechanism of permeation in PV and RO, in which permeants dissolve into a membrane, and then diffuse through it down a concentration gradient. According to the SD model, the flux in PV is usually given as $J_{i}$, as follows [29]:

(4) $$J_{i}=\frac{P_{i}}{\ell }\left(p_{io}-p_{i1}\right)$$

where ℓ is the membrane thickness and $p_{io}$ and $p_{i1}$ are the partial pressures of component i on feed surface and permeate surface of the membrane, respectively; $P_{i}$ is the membrane permeability, given as:

(5) $$P_{i}=D_{i}K_{i}=J_{i}\frac{\ell }{p_{io}-p_{i1}}$$

where $D_{i}$ and $K_{i}$ are the diffusion coefficient and sorption coefficient of component i, respectively. When the membrane thickness is unknown, membrane permeance ($\frac{P_{i}}{\ell }$), a component flux normalized for driving force can be used, expressed as follows:

(6) $$\frac{P_{i}}{\ell }=\frac{D_{i}K_{i}}{\ell }=\frac{J_{i}}{p_{io}-p_{i1}}$$

Under the PV desalination conditions, since the salt ions cannot evaporate, $p_{io}$ and $p_{i1}$ were simplified to the vapor pressures of water on feed side, $p_{\mathrm{sat}}$, and on permeate side, $p_{2}$, respectively. When the vapor pressure on the permeate $p_{2}$ is often assumed to be zero, a simplified expression for water permeance $P_{w}$ can then be written as:

(7) $$P_{w}=\frac{J_{v}}{p_{\mathrm{sat}}}$$

The volume flux of water $J_{v}$, can be expressed as follows:

(8) $$J_{v}=\frac{V}{A\cdot \Delta t}$$

where V is the volume of water collected at the experimental time interval Δt, and A is the effective membrane area. The saturated vapor pressure of water on feed side, $p_{\mathrm{sat}}$, can be calculated using the Antoine Equation as follows:

(9) $$p_{\mathrm{sat}}\;=exp\left(A-\frac{B}{C+T}\right)$$

where A, B, and C are Antoine constants, and T is the absolute temperature.

The observed salt rejection, $R_{\mathrm{obs}}$, can be expressed as follows:

(10) $$R_{\mathrm{obs}}=\left(1-\frac{C_{p}}{C_{f}}\right)\times 100\%$$

where $C_{p}$ and $C_{f}$ are the concentrations of the permeate and the feed, respectively, which were measured using an ion chromatography (Metrohm Eco IC).

## 3. Results and Discussions

### 3.1. Characterization of the Membranes

**Figure 1:**
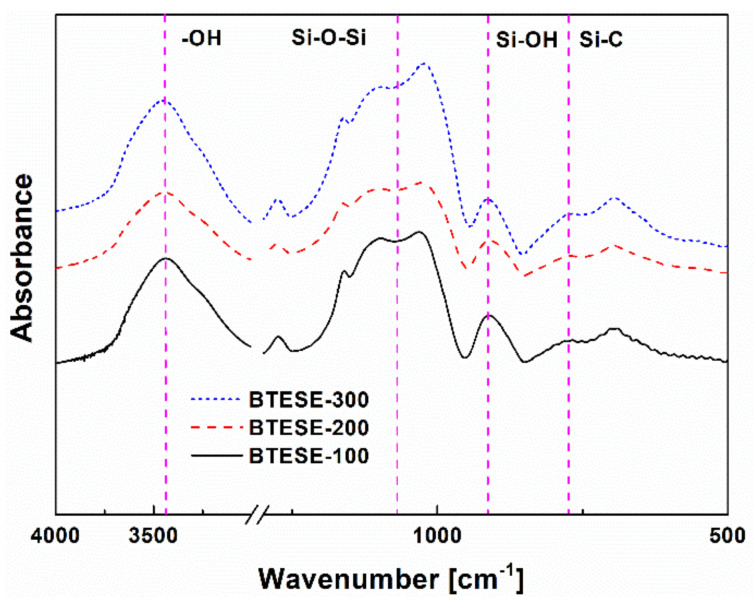
Fourier transform infrared spectrometer (FT-IR) spectra of the 1,2-bis(triethoxylsilyl)ethane (BTESE) films fired at 100, 200, and 300 °C, respectively.

Figure 1 shows the FT-IR absorbance spectra of BTESE-derived films calcined at 100, 200, and 300 °C under air atmosphere, respectively. The absorption band around 920 cm^–1^ was ascribed to Si–OH stretch and the peak at ~790 cm^–1^ was assigned to a Si–C stretch. The characteristic bands between 1020 and 1200 cm^–1^ corresponded to stretching vibrations of Si–O–Si groups. Moreover, a broad band that appeared in the region from 3000 to 3600 cm^–1^ could be assigned to an O–H stretch [30,31]. To investigate the effect of calcination temperatures on the condensation of BTESE networks, the peak area ratio of Si–OH/Si–O–Si was calculated as indicators of the condensation degree of Si–OH to Si–O–Si groups in the networks. As shown in Table 1, it was clear that the ratio of the peak area decreased with an increase in the calcination temperature due to the further condensation reaction of the silanol groups, which led to the development of less hydrophilic surface chemistry. This agreed well with the results of water contact angle measurements, which also showed a gradual increased surface hydrophobicity of the BTESE films upon increasing the calcination temperature (Table 1). 

**Figure 2:**
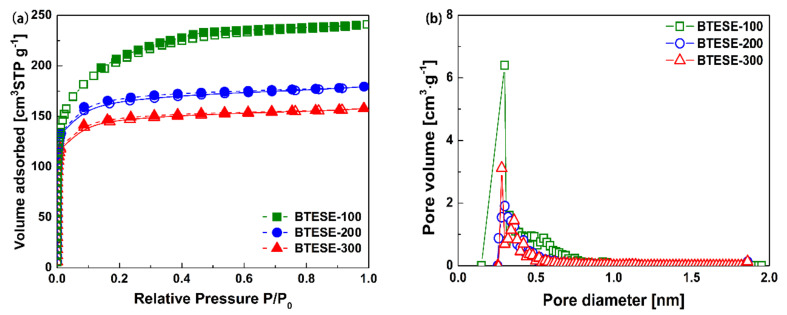
(**a**) N_2_ adsorption-desorption isotherms (solid: adsorption, dot: desorption) and (**b**) pore size distribution (calculated by MP method) of BTESE xerogels with different calcination temperatures.

To examine the pore structure, N_2_ sorption measurements were carried out on the BTESE xerogels, which were prepared by heat-treatment at 100, 200, and 300 °C, respectively. As presented in Figure 2a, all N_2_ isotherms showed type-I characteristics with a significant uptake of N_2_ at low relative pressure region (P/P_0_ < 0.1), which is typical of microporous materials [32]. On the other hand, all samples showed narrow pore size distributions with nominal pore diameters centered at approximately 0.3 nm (Figure 2b), probably due to the identical organic bridges in the silica networks. Details of the textural properties are summarized in Table 2. It was clear that, from BTESE–100 to BTESE–300, the BET specific surface area decreased gradually. This can be ascribed to the changes in pore structures of the three samples. As confirmed by FT-IR analysis, a higher calcination temperature would accelerate the dehydroxylation reaction of silanol groups and the formation of siloxane bonds, thus resulting in a denser organosilica network.

**Figure 3:**
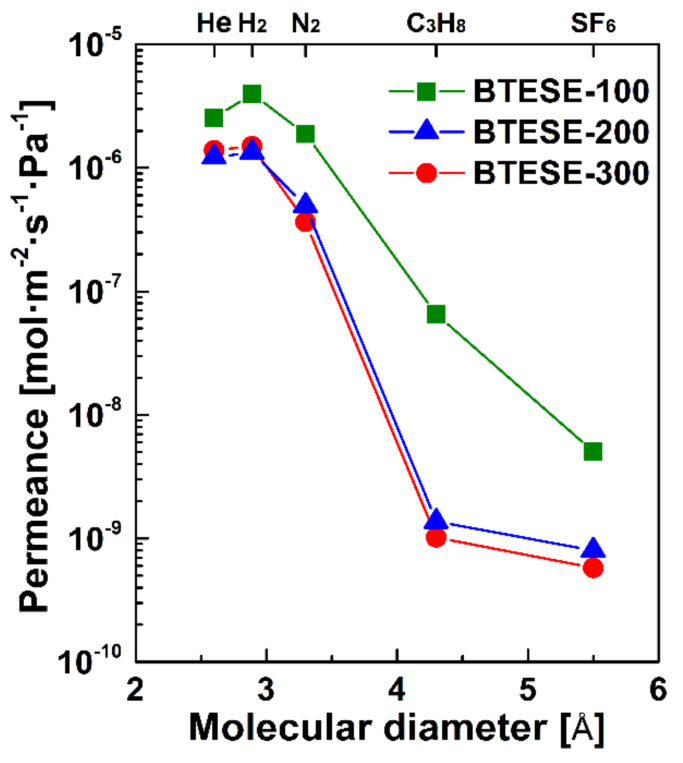
Gas permeation properties of BTESE-100, BTESE-200, and BTESE-300 membranes as a function of molecular size tested at 100 °C.

Figure 3 shows single gas permeation performances of the three membranes with different calcination temperatures. The H_2_ permeance with H_2_/N_2_ and H_2_/SF_6_ permeance ratios are listed in Table 3. In general, the BTESE-100 membrane exhibited higher permeance and lower permeance ratios of H_2_/N_2_ and H_2_/SF_6_ than the other two membranes, suggesting a looser network structure. On the other hand, the gas permeation performances were similar for BTESE-200 and BTESE-300 membranes. Both membranes showed high H_2_ permeances (approximately 10^−6^ mol m^−2^ s^−1^ Pa^−1^) with moderate permeance ratios of 2.7–4.1 for H_2_/N_2_ and high ratios of 1670–2590 for H_2_/SF_6_. In addition, all BTESE membranes showed a higher permeance for H_2_ than that for He, despite the larger molecular size of H_2_. This can be explained by Knudsen diffusivity, in which H_2_ with a smaller molecular weight leads to a higher diffusivity. For N_2_, C_3_H_8_, and SF_6_ molecules, the transport mechanism through BTESE membranes is governed by molecular sieving [33], where molecules with a larger size show a lower permeance. The membrane fabricated at high temperatures showed a decrease in gas permeances with an enhanced H_2_ selectivity due to the densification of organosilica networks.

**Figure 4:**
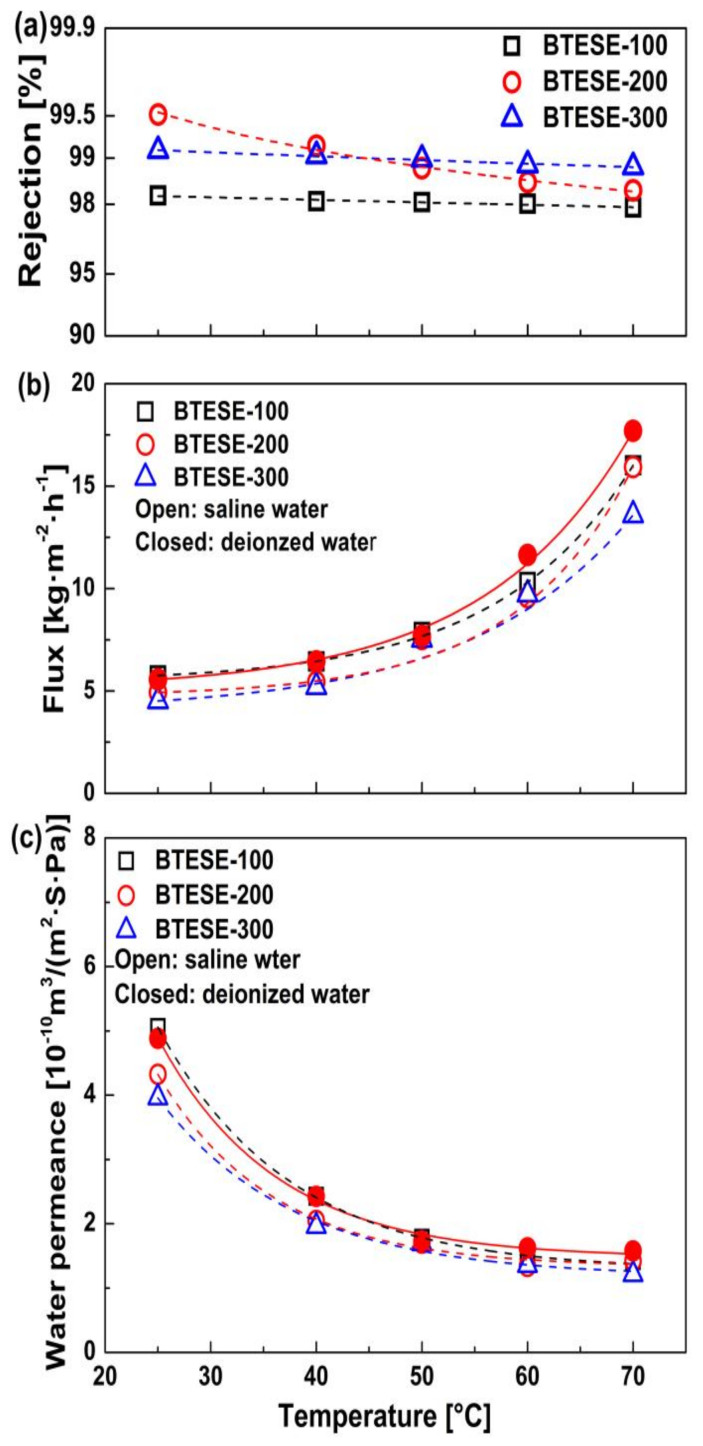
Temperature dependence of (**a**) NaCl rejection, (**b**) water flux, and (**c**) water permeance for the BTESE membranes at a feed concentration of 2000 ppm NaCl (closed circle: feeding with deionized water using the BTESE-200 membrane).

The pore sizes of the BTESE membranes could be further quantitatively determined by using the Normalized-Knudsen-based Permeance (NKP) method, as shown in Figure 4, and the results are given in Table 3. The NKP equation, based on the modified-GT model, can be expressed as follows [34]:

(11) $$f_{NKP}=\frac{P_{i}}{P_{s}}\sqrt{\frac{M_{i}}{M_{s}}}\approx \frac{{\left(1-\frac{d_{i}}{d_{p}}\right)}^{3}}{{\left(1-\frac{d_{s}}{d_{p}}\right)}^{3}}$$

where *d_p_* is the mean effective pore size, *d_i_* and *d_s_* are the dynamic diameter, while *P_i_* and *P_s_* are the permeance of gas *i* and *s*.

As presented in Table 3, the calculated pore size of the membranes decreased from 0.56 to 0.53 nm as the calcination temperature increased from 100 to 300 °C. The small difference in pore size for these membranes showed little effect on the selectivity of H_2_/N_2_ but obvious difference in the selectivity of H_2_/SF_6_. This is due to smaller kinetic diameter of H_2_ and N_2_ (0.289 and 0.364 nm), compared to the membrane pore size. The kinetic diameter of SF_6_ (0.55 nm), however, is very close to the membrane pore size. Thus, a large difference in permeance ratios of H_2_/SF_6_ was observed.

### 3.2. Pervaporation Performance for Desalination

The effect of calcination temperature on PV desalination performances for the BTESE membranes has been systemically evaluated. Figure 4 shows the performances of BTESE-100, BTESE-200, and BTESE-300 as a function of operating temperatures. Compared with BTESE-200 and BTESE-300 membranes, the BTESE-100 membrane showed the highest water flux and permeance with the lowest NaCl rejection regardless of the operating temperature, which is similar to the results of single gas permeation. The IR analysis confirmed that the number of silanol groups in BTESE-100 networks was larger than that in BTESE-200 and BTESE-300 networks. Meanwhile, a larger pore size was obtained for the BTESE-100 networks by a lower calcination temperature. The loose network structure together with much hydrophilic silanol groups in the BTESE-100 networks would lead to a higher water adsorption and diffusivity, resulting in the highest water flux and permeance. However, the larger pore size would allow more hydrated salt ions to pass through the membrane, thus reducing the salt rejection. It is noteworthy that the water flux for all membranes was increased gradually with increasing the operation temperature due to the increased driving force for water transport (Figure 4b). In contrast, the water permeance decreased gradually as the temperature increased (Figure 4c). According to the solution-diffusion model, the permeance (*P*) can be expressed as the product of diffusivity (*D*) and solubility (*K*), as given by Equation (5). Diffusivity almost always increases with increasing temperature, whereas the sorption normally decreases. This indicates that the change in sorption outweighs the change in diffusivity during the water permeation, thus resulting in a decrease in the water permeance.

The temperature dependence of the water flux (J) in pervaporation generally follows the Arrhenius equation:

(12) $$J=A_{0}exp(-\frac{Eapp}{RT})$$

where $A_{0}$ is the pre-exponential factor, $E_{app}$ is the apparent activation energy of the permeation, R is the gas constant, and T is the absolute temperature. Table 4 summarized the apparent activation energy for the membranes with different calcination temperatures. The highest value of $E_{app}$ was observed for the BTESE-300 membrane due to the smallest pore size of the membrane. Deionized water was also used as feed to illustrate the effect of hydrated salt ions on the mass transport through the membrane. The flux, permeance, and apparent activation energy of pure water through the BTESE-200 membrane are shown in Figure 4b, Figure 4c (solid red circle), and Table 4. The flux for pure water feed increased from 5.56 to 17.7 kg∙m^−2^∙h^−1^ in the temperature range of 25–70 °C, which is higher than that for the aqueous salt solution (from 4.93 to 15.9 kg∙m^−2^∙h^−1^). Similarly, the apparent activation energy for pure water permeation through the BTESE-200 membrane is lower than that for the NaCl aqueous solution.

**Figure 5:**
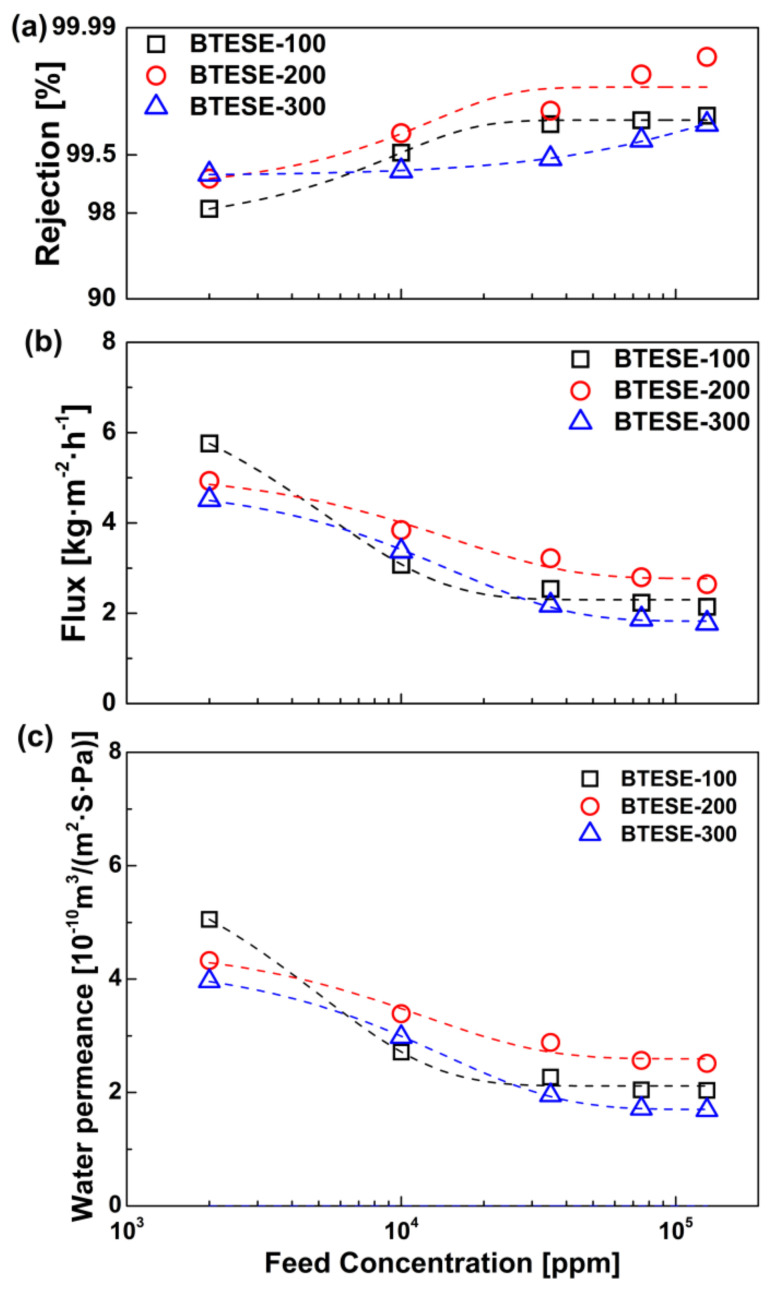
Effect of feed concentration on (**a**) NaCl rejection, (**b**) water flux, and (**c**) water permeance of the BTESE membranes at 25 °C.

Figure 5 shows the effect of feed salinity on permeation performances of the BTESE membranes at an operating temperature of 25 °C. Generally, both water flux and water permeance of the three membranes decreased gradually with an increase in feed concentration. Since a change in the feed concentration directly affected sorption at the interface between the feed solution and the membrane [35], the concentration of adsorbed hydrated ions on the membrane increased upon increasing the feed concentration, thus simultaneously lowering the water flux and water permeance. Meanwhile, the saturated vapor pressure of the feed solutions, *P*_sat_, was decreased slightly with an increase in the NaCl concentration, which is indicative of a decrease in driving force for water permeation. Contrary to this trend, the NaCl rejection even showed a slight increase with an increase in the feed concentration. Since the average pore size of these BTESE membranes ranged from 0.53 to 0.56 nm, the larger hydrated salt ions (Na^+^_(aq)_: 0.72 nm and Cl^−^_(aq)_: 0.66 nm) would be perfectly rejected due to the molecular sieving effect [36]. Therefore, the increase in observed salt rejection was mainly due to the increased feed concentrations *C*_f_, as presented by Equation (9). It should be noted that compared with the BTESE-200 membrane, a greater drop in water flux was observed for the BTESE-100 membrane with an increase in feed concentration, likely due to its structural instability in harsh high-salinity water environments. Similar structural degradation has been observed for the ZSM-5 membranes in PV desalination of high-salinity water [16]. In addition, the highest rejection was achieved with the BTESE-200 membrane instead of the BTESE-300 membrane as the salt concentration increased, possibly due to the difference in Si–OH density in the two silica networks. Compared with BTESE-300 membrane, a higher density of Si–OH groups was obtained in BTESE-200 networks, which contributed to a faster transport of water molecules through the membrane. The more rapid transport of water would to a lower concentration of permeate, and therefore, a slightly higher observed salt rejection, assuming the same salt flux for the two membranes. However, the BTESE networks are amorphous in nature, leading to a relatively broad pore size distribution. Therefore, it is possible that a small number of hydrated salt ions could penetrate as liquid through the large pores and/or small defects in the membrane, resulting in an incomplete rejection of hydrated ions (>98.2%).

**Figure 6:**
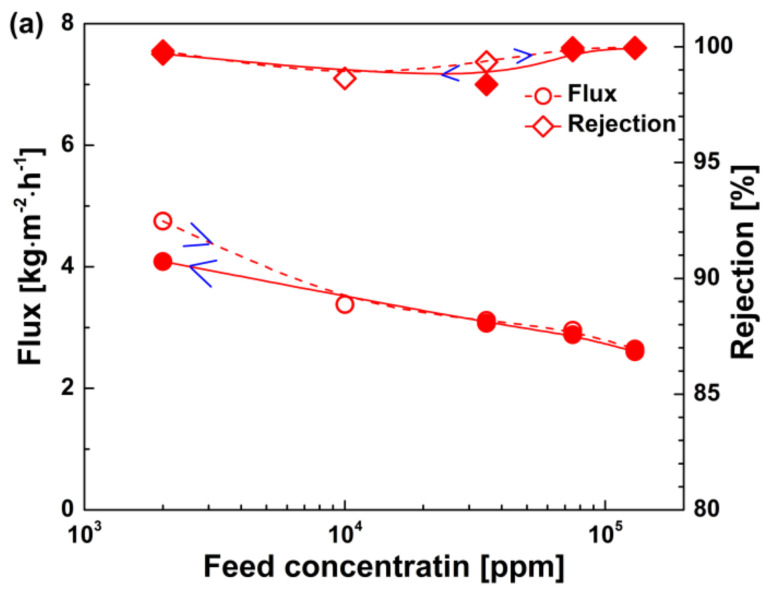

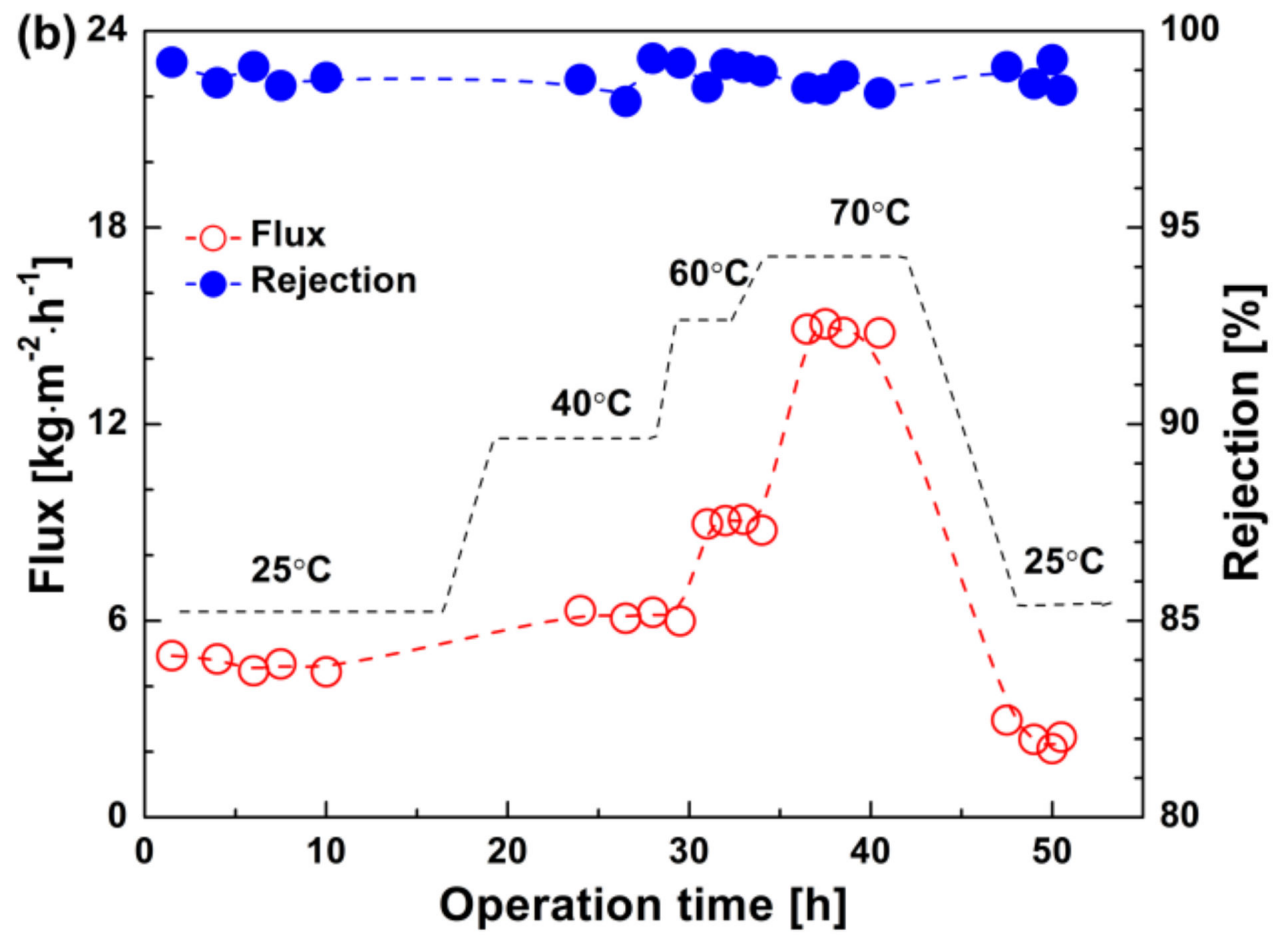
Water flux and NaCl rejection of the BTESE-200 membrane as a function of (**a**) concentration cycles and (**b**) temperature cycles.

Figure 6 shows water flux and NaCl rejection of the BTESE-200 membrane as a function of concentration and temperature cycles. As feed concentration increased from 2000 to 130,000 ppm and then decreased gradually back to the starting level, water flux could returned to approximately the initial value, and high salt rejection was maintained (Figure 6b). Similarly, in the temperature cycles of 25 to 70 °C, the NaCl rejection was always in excess of 98%, irrespective of the operating temperature. However, when the feed temperature decreased back to 25 °C, the water flux of the membrane reduced to ~2.5 kg∙m^−2^∙h^−1^, which is obviously lower than its initial value of ~4.5 kg∙m^−2^∙h^−1^ at 25 °C. The decrease in water flux in the temperature cycles is probably due to the fouling of the membrane surface and pores.

**Figure 7:**
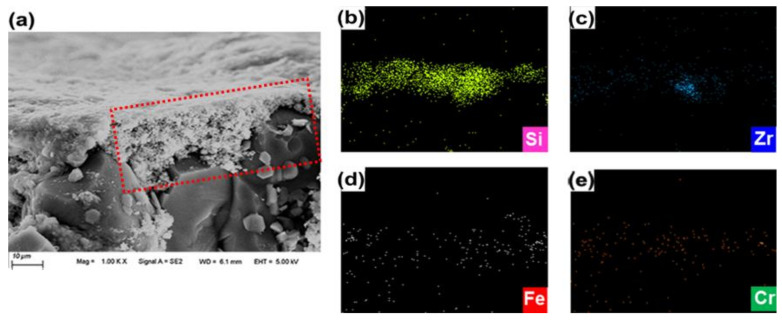
SEM (**a**) and EDX (**b**–**e**) images of cross-section of the BTESE-200 membrane after a continuous PV operation of 50 h.

Figure 7 shows the results of the energy dispersive X-ray (EDX) and SEM analysis on the BTESE-200 membrane after a continuous PV desalination of 50 h. The elements of Si, Zr, Fe, and Cr were observed in the membrane sample. Since Fe and Cr were not the composition of the BTESE membrane, they were probably from the stainless steel membrane module and dissolved in the aqueous salt solution. These metal ions can form colloidal hydroxide particles with assistance of dissolved oxygen in water, which would cause the fouling of membrane surface and pores and result in a decrease in membrane flux.

## 4. Conclusions

BTESE-derived organosilica membranes were fabricated and applied to water desalination. The structural and surface properties of the BTESE networks were finely tailored by changing the calcination temperatures. The desalination performances and transport properties of these membranes were extensively investigated. The surface hydrophilicity of the BTESE networks decreased with an increase in calcination temperature due to the enhanced condensation of Si–OH groups. The pore size of the membranes decreased as the calcination temperature increased, which were evaluated quantitatively using the Normalized Knudsen-based permeance (NKP) model. In pervaporation tests, higher calcination temperature would lead to higher salt rejection and lower water flux/permeance, which was ascribed to the changes in pore size and surface chemistry of BTESE networks. Moreover, the BTESE membranes calcined at 200 °C showed high reproducibility in NaCl concentration cycles of 0.2–13 wt% and superior hydrothermal stability in temperature cycles up to 70 °C, showing great potential in desalination applications.

## Figures and Tables

**Table 1:** Water contact angles and FT-IR peak area ratios of Si–OH to Si–O–Si of the BTESE films fabricated at different temperatures.

Samples	Peak Area Ratios (Si–OH/Si–O–Si)	Contact Angles (°)
BTESE-100	0.53	52.0 ± 0.5
BTESE-200	0.48	56.4 ± 0.5
BTESE-300	0.41	68.0 ± 0.5

**Table 2:** Textural properties of the samples with different calcination temperatures.

Samples	*S*_BET_ [m^2^ g^−1^]	*V*_p_ [cm^3^ g^−1^]	*D*_p_ [nm]
BTESE-100	728	0.16	0.32
BTESE-200	532	0.18	0.32
BTESE-300	472	0.17	0.30

**Table 3:** Gas permeation performances and Normalized Knudsen-based permeance (NKP) pore sizes of the BTESE membranes.

Membranes	*P*_H2_ [mol m^−2^ s^−1^ Pa^−1^]	H_2_/N_2_	H_2_/SF_6_	NKP Pore Size (nm)
BTESE-100	3.98 × 10^−6^	2.1	790	0.56
BTESE-200	1.34 × 10^−6^	2.7	1670	0.54
BTESE-300	1.50 × 10^−6^	4.1	2590	0.53

**Table 4:** Apparent activation energy *E_app_* for water permeation through the BTESE membranes.

Samples	Saline Water (2000 ppm NaCl)			Deionized Water BTESE-200
	BTESE-100	BTESE-200	BTESE-300	
***E_app_* (kJ∙mol^−1^)**	12.32	14.99	15.49	14.08

## References

[1] Elimelech M., Phillip W.A. (2011). The future of seawater desalination: Energy, technology, and the environment. Science.

[2] Greenlee L.F., Lawler D.F., Freeman B.D., Marrot B., Moulin P. (2009). Reverse osmosis desalination: Water sources, technology, and today’s challenges. Water Res..

[3] Mansouri J., Harrison S., Chen V. (2010). Strategies for controlling biofouling in membrane filtration systems: Challenges and opportunities. J. Mater. Chem..

[4] Lawson K.W., Lloyd D.R. (1997). Membrane distillation. J. Membr. Sci..

[5] Sadrzadeh M., Mohammadi T. (2008). Sea water desalination using electrodialysis. Desalination.

[6] McCutcheon J.R., McGinnis R.L., Elimelech M. (2005). A novel ammonia-carbon dioxide forward (direct) osmosis desalination process. Desalination.

[7] Feng X.S., Huang R.Y.M. (1997). Liquid separation by membrane pervaporation: A review. Ind. Eng. Chem. Res..

[8] Subramani A., Jacangelo J.G. (2015). Emerging Desalination Technologies for Water Treatment: A Critical Review. Water Res..

[9] Wang Q., Li N., Bolto B., Hoang M., Xie Z. (2016). Desalination by pervaporation: A review. Desalination.

[10] Shaffer D.L., Chavez L.H.A., Ben-Sasson M., Romero-Vargas Castrillón S., Yip N.Y., Elimelech M. (2013). Desalination and reuse of high-salinity shale gas produced water: Drivers, technologies, and future directions. Environ. Sci. Technol..

[11] Kaminski W., Marszalek J., Tomczak E. (2018). Water desalination by pervaporation—Comparison of energy consumption. Desalination.

[12] Xue Y.L., Huang J., Lau C.H., Cao B., Li P. (2020). Tailoring the molecular structure of crosslinked polymers for pervaporation desalination. Nat. Commun..

[13] Song Y.M., Li R., Pan F.S., He Z., Yang H., Li Y., Yang L.X., Wang M.D., Wang H.J., Jiang Z.Y. (2019). Ultrapermeable graphene oxide membranes with tunable interlayer distances via vein-like supramolecular dendrimers. J. Mater. Chem. A.

[14] Zhu Y.Q., Gupta K.M., Liu Q., Jiang J.W., Caro J., Huang A.S. (2016). Synthesis and seawater desalination of molecular sieving zeolitic imidazolate framework membranes. Desalination.

[15] Duke M.C., Abrahamb J.O., Milnea N., Zhu B., Lin J.Y.S., Diniz da Costa J.C. (2009). Seawater desalination performance of MFI type membranes made by secondary growth. Sep. Sci. Technol..

[16] Drobek M., Yacou C., Motuzas J., Julbe A., Ding L.P., Diniz da Costa J.C. (2012). Long term pervaporation desalination of tubular MFI zeolite membranes. J. Membr. Sci..

[17] Cao Z.S., Zeng S.X., Xu Z., Arvanitis A., Yang S.W., Gu X.H., Dong J.H. (2018). Ultrathin ZSM-5 zeolite nanosheet laminated membrane for high-flux desalination of concentrated brines. Sci. Adv..

[18] Castricum H.L., Sah A., Kreiter R., Blank D.H.A., Vente J.F., ten Elshof J.E. (2008). Hybrid Ceramic Nanosieves: Stabilizing Nanopores with Organic Links. Chem. Commun..

[19] Castricum H.L., Sah A., Kreiter R., Blank D.H.A., Vente J.F., ten Elshof J.E. (2008). Hydrothermally Stable Molecular Separation Membranes from Organically Linked Silica. J. Mater. Chem..

[20] Kanezashi M., Yada K., Yoshioka T., Tsuru T. (2008). Design of silica networks for development of highly permeable hydrogen separation membranes with hydrothermal stability. J. Am. Chem. Soc..

[21] Xu R., Wang J., Kanezashi M., Yoshioka T., Tsuru T. (2011). Development of robust organosilica membranes for reverse osmosis. Langmuir.

[22] Pizzoccaro-Zilamy M.A., Huiskes C., Keim E.G., Sluijter S.N., van Veen H., Nijmeijer A., Winnubst L., Luiten-Olieman M.W.J. (2019). New Generation of Mesoporous Silica Membranes Prepared by a Stöber-Solution Pore-Growth Approach. ACS Appl. Mater. Interfaces.

[23] Ciriminna R., Fidalgo A., Pandarus V., Beland F., Ilharco L.M., Pagliaro M. (2013). The sol-gel route to advanced silica-based materials and recent applications. Chem. Rev..

[24] Niimi T., Nagasawa H., Kanezashi M., Yoshioka T., Ito K., Tsuru T. (2014). Preparation of BTESE-derived organosilica membranes for catalytic membrane reactors of methylcyclohexane dehydrogenation. J. Membr. Sci..

[25] Song H., Wei Y., Wang C., Zhao S., Qi H. (2018). Tuning sol size to optimize organosilica membranes for gas separation. Chin. J. Chem. Eng..

[26] Kanezashi M., Sasaki T., Tawarayama H., Nagasawa H., Yoshioka T., Ito K., Tsuru T. (2014). Experimental and theoretical study on small gas permeation properties through amorphous silica membranes fabricated at different temperatures. J. Phys. Chem. C.

[27] Song H., Wei Y., Qi H. (2017). Tailoring pore structures to improve the permselectivity of organosilica membranes by tuning calcination parameters. J. Mater. Chem. A.

[28] Xu R., Zou L., Lin P., Zhang Q., Zhong J. (2016). Pervaporative desulfurization of model gasoline using PDMS/BTESE-derived organosilica hybrid membranes. Fuel Process. Technol..

[29] Wijmans J.G., Baker R.W. (1995). The solution-diffusion model: A review. J. Membr. Sci..

[30] Ngamou P.H.T., Overbeek J.P., Kreiter R., van Veen H.M., Vente J.F., Wienk I.M., Cuperus P.F., Creatore M. (2013). Plasma-Deposited Hybrid Silica Membranes with a Controlled Retention of Organic Bridges. J. Mater. Chem. A.

[31] Xu R., Ibrahim S.M., Kanezashi M., Yoshioka T., Ito K., Ohshita J., Tsuru T. (2014). New Insights into the Microstructure-Separation Properties of Organosilica Membranes with Ethane, Ethylene, and Acetylene Bridges. ACS Appl. Mater. Interfaces.

[32] Fierro V., Szczurek A., Zlotea C., Marêché J.F., Izquierdo M.T., Albiniak A., Latroche M., Furdin G., Celzard A. (2010). Experimental evidence of an upper limit for hydrogen storage at 77 K on activated carbons. Carbon.

[33] Tsuru T., Igi R., Kanezashi M., Yoshioka T., Fujisaki S., Iwamoto Y. (2011). Permeation properties of hydrogen and water vapor through porous silica membranes at high temperatures. AIChE J..

[34] Nagasawa H., Niimi T., Kanezashi M., Yoshioka T., Tsuru T. (2014). Modified gas-translation model for prediction of gas permeation through microporous organosilica membranes. AIChE J..

[35] Xu R., Lin P., Zhang Q., Zhong J., Tsuru T. (2016). Development of ethenylene-bridged organosilica membranes for desalination applications. Ind. Eng. Chem. Res..

[36] Zhou M., Nemade P.R., Lu X., Zeng X., Hatakeyama E.S., Noble R.D., Gin D.L. (2007). New type of membrane material for water desalination based on a cross-linked bicontinuous cubic lyotropic liquid crystal assembly. J. Am. Chem. Soc..

